# Carbon nanotube biosensors

**DOI:** 10.3389/fchem.2015.00059

**Published:** 2015-10-27

**Authors:** Carmen-Mihaela Tîlmaciu, May C. Morris

**Affiliations:** Cell Cycle Biosensors and Inhibitors, Faculté de Pharmacie, Institut des Biomolécules Max Mousseron, Centre National de la Recherche Scientifique-UMR 5247Montpellier, France

**Keywords:** carbon nanotube, biosensing, fluorescence, functionalization, biocompatibility, internalization, cancer

## Abstract

Nanomaterials possess unique features which make them particularly attractive for biosensing applications. In particular, carbon nanotubes (CNTs) can serve as scaffolds for immobilization of biomolecules at their surface, and combine several exceptional physical, chemical, electrical, and optical characteristics properties which make them one of the best suited materials for the transduction of signals associated with the recognition of analytes, metabolites, or disease biomarkers. Here we provide a comprehensive review on these carbon nanostructures, in which we describe their structural and physical properties, functionalization and cellular uptake, biocompatibility, and toxicity issues. We further review historical developments in the field of biosensors, and describe the different types of biosensors which have been developed over time, with specific focus on CNT-conjugates engineered for biosensing applications, and in particular detection of cancer biomarkers.

## History and introduction to carbon nanotubes

Brought to our planet from the red giant stars, carbon is a singular element in the periodic table: it can bind itself or other atoms without a great expense of energy. Fundamental for the living world, carbon has long been known to exist in three allotropic forms: graphite, diamond, and amorphous carbon. After the Second World War, in the middle of the last century, tremendous progress in the science of carbon led to unexpected and fascinating findings. For example lonsdaleite, also called hexagonal diamond, was first identified in 1967 from the Canyon Diablo meteorite, where it occurred as microscopic crystals associated with diamond. Later, the discovery of buckminsterfullerenes (C_60_) (Kroto et al., 1985) marked the beginning of a new era in carbon science. The impact was so huge that its discoverers, Robert Curl Jr., Harold Kroto and Richard Smalley were awarded the Nobel Prize in Chemistry in 1996.

Here we will focus on carbon nanotubes (CNTs), also called buckytubes and first evidenced in 1991 by the Japanese electron microscopist Sumio Iijima. Since his first report on multi-walled carbon nanotubes (MWNTs) (Iijima, 1991), followed by their single-walled counterparts (SWNTs) (Iijima and Ichihashi, 1993; Journet et al., 1997), CNTs have emerged as one of the most intensively investigated nanostructured materials (Balasubramanian and Burghard, 2005), with thousands of papers published every year, offering promises for new applications which have attracted both academic and industrial interest. CNTs are hollow carbon structures, with one or more walls, a nanometer scale diameter and a comparatively more important length. They exhibit a well-ordered arrangement of carbon atoms linked via sp^2^ bonds, which makes them the stiffest and strongest fibers known. Their advantage compared to other nanomaterials lies in a unique combination of electrical, magnetic, optical, mechanical, and chemical properties, which offer great promises for a wide range of applications, including biosensing (Le Goff et al., 2011; Biju, 2014).

Besides, CNTs can serve as platforms to conjugate other compounds at their surface (exohedral functionalization) (Arkan et al., 2015; Shobha and Muniraj, 2015). Moreover, CNTs shells can be opened and filled (endohedral functionalization) without losing their stability (Sloan et al., 1998).

Importantly, functionalized CNTs can effectively cross biological barriers such as the cell membrane and penetrate individual cells (Pantarotto et al., 2004b). This feature and the mechanism of internalization and release of CNTs from the cells are of major interest for biological and in particular intracellular biosensing applications.

Generally, the successful application of CNTs for biomedical tests faces a variety of challenges prerequisites. These include: (a) synthesis of CNTs with tailored functionalities and uniform morphology; (b) modification of CNTs to make them compatible with biological systems; (c) detailed study of their interaction with biological environments (toxicity, interaction with single cells); (d) *in vivo* testing for specific therapeutic and diagnostic purposes such as imaging (contrast agents, markers), sensing (nanoparticle-based diagnostics) and cancer treatment (hyperthermia, drug delivery).

### Structure and properties

**Figure 1:**
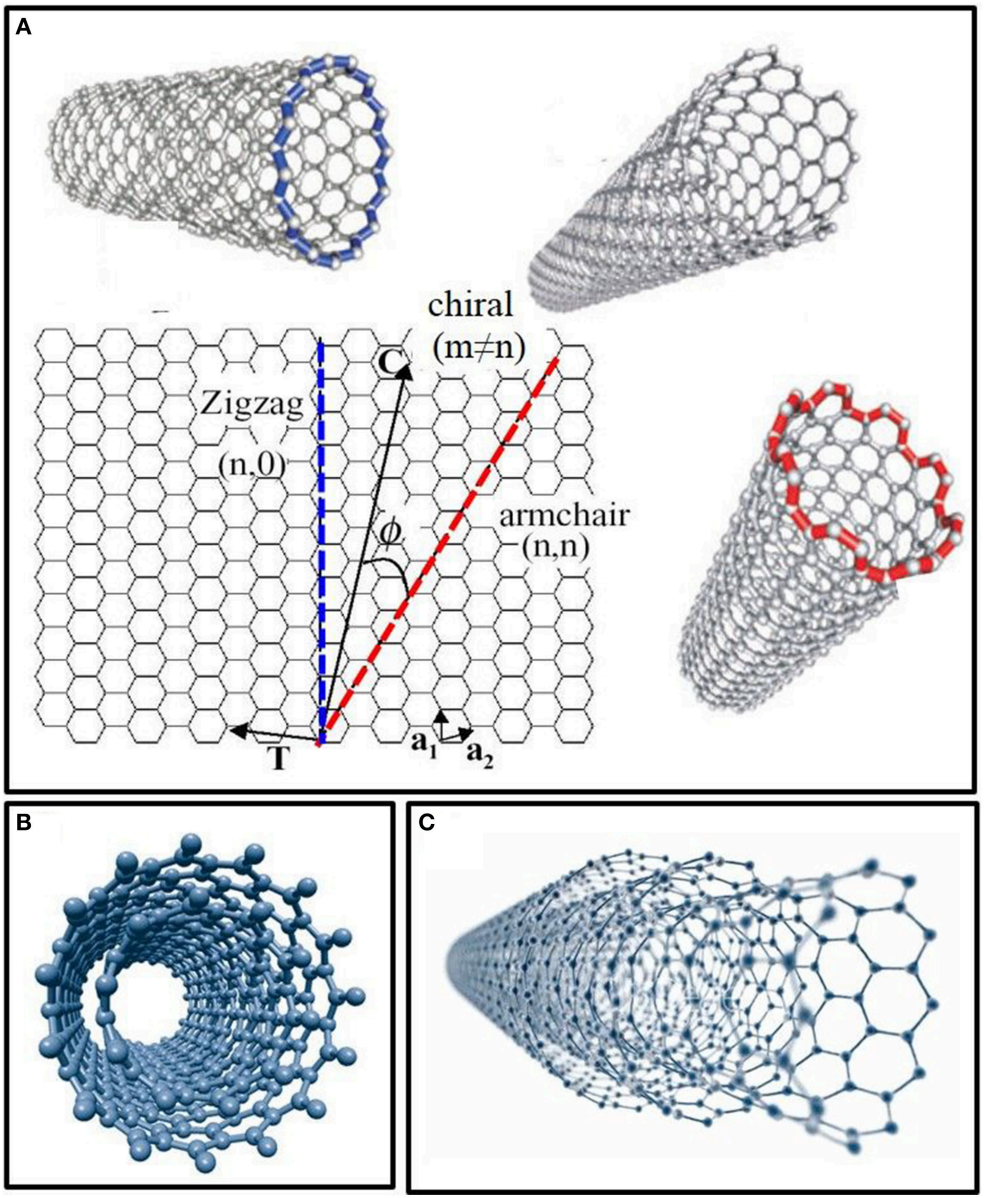
**Structure and models of carbon nanotubes in function of their number of walls. (A)** Single-walled carbon nanotubes (SWNTs) structures in function of their chirality (zigzag, armchair, and chiral). **(B)** Model of double-walled carbon nanotubes (DWNTs). **(C)** Structure of multi-walled carbon nanotubes (MWNTs) made up of several concentric shells.

Depending on their number of walls, CNTs are designated single-walled (SWNTs) or multi-walled (MWNTs). Cylinder-shaped SWNTs with diameters as small as a nanometer (or less) can be grown up to 20 cm in length (Zhu et al., 2002). The side-walls of these tubes are made up of a hexagonal lattice of carbon atoms, similar to the atomic planes of graphene and are usually capped at both ends by one half of a fullerene-like molecule. SWNTs possess the simplest morphology and can be visualized as a single rolled up graphene sheet. Based on the orientation of the tube axis with respect to the hexagonal lattice, the structure of a nanotube can be simply defined through its chiral vector, which is defined by the chiral indices (*n, m*). SWNTs are classified by the geometric arrangement of the carbon atoms at the seam of the cylinders. While most SWNTs are chiral (*m* ≠ *n*), some of them present armchair (*m* = *n*) or zigzag (*m* = 0) configurations (Figure 1A).

In the most general case, a CNT is composed of a concentric arrangement of several cylinders (Figure 1C). Such MWNTs can reach diameters of up to 100 nm and the distance between two walls is very close to the distance between two graphene layers in graphite (~3.5 Å) (Balasubramanian and Burghard, 2005). Double-walled carbon nanotubes (DWNTs) are a special case of MWNTs, composed of just two concentric cylinders (Figure 1B). DWNTs bridge the gap between SWNTs and MWNTs, thereby cumulating properties of both kinds of CNTs. More specifically, DWNTs resemble SWNTs with respect to their small diameter, length and ability to form bundles, but their mechanical stability is much greater than that of the SWNTs, especially when covalently functionalized (Yang et al., 2010). Moreover, the outer wall of DWNTs can be functionalized without affecting the mechanical and electrochemical properties of the inner tube, just like MWNTs (Pumera, 2007).

Besides, CNTs have a large specific surface area (SSA), which enables immobilization of a large number of functional units at the carbon nanotube surface, such as receptor moieties for biosensing applications. In practice, the bundling effect, as well as the increase in the number of walls, decreases the SSA of CNTs (Peigney et al., 2001). It is worth noting that the properties of CNTs may differ significantly between MWNTs and SWNTs. SWNTs are unique nanostructures with unusual electronic properties, because of the one-dimensional quantum effect. Depending on their diameter and chirality, CNTs may be either semi-conducting or semi-metallic (Hamada et al., 1992; Saito and Yoshikawa, 1993). For example, the armchair structure behaves as a metallic material, whereas the zigzag structure has semi-conductor or quasi-metallic properties. In the latter case, the width of the band gap of the semi-conductor decreases with the increase of CNT diameter (Mintmire and White, 1995). Two properties are responsible for the high electrical conductivity of metallic CNTs: they have very few defects to scatter electrons and they present a good stability at high temperatures (up to 300°C in air and 1500°C in vacuum). Hence, a good ballistic conduction is also detected (Frank et al., 1998). Moreover, their mechanical properties are excellent, combining high strength with high stiffness. The tensile strength of SWNTs is about 20 times that of steel (Yu et al., 2000) and the Young's modulus of CNTs is much greater than that of steel fibers. CNTs may also present a positive or negative magnetoresistance, as a function of the temperature and the applied magnetic field. For example, in a weak magnetic field, nanotubes exhibit large diamagnetic and paramagnetic responses, depending on the field direction, Fermi energy, helicity, and size of the nanotubes (Lu, 1995).

Owing to their quasi 1-D nature, SWNTs exhibit strong resonance Raman scattering, high optical absorption and photoluminescence in the Near Infra-Red (NIR) range, properties that present a high interest for imaging in biological systems *in vitro* and *in vivo* (Zhou et al., 2009). The most important feature in the Raman spectrum of CNTs is the Radial Breathing Mode (RBM), which is often located between 100 and 250 cm^−1^, providing information about the CNT diameters (Michel et al., 2009). The increase of the RBM frequency indicates that the CNTs agglomerate in bundles. Then, two bands are located between 1300 and 1700 cm^−1^: the D band, at about 1320 cm^−1^ is due to the phonons induced by the crystalline disorder and it gives information about the CNT structural defects (sp^3^ carbon); the G band, at about 1580 cm^−1^, is characteristic of the carbon sp^2^ hybridization. The ratio of these two bands intensities (I_D_/I_G_) gives general information about the structural quality of the CNT. The I_D_/I_G_ ratio increases with the level of defects on the CNT surface. Another band appears at 2600 cm^−1^, the G'_2D_ band, which confirms the presence of CNTs in the biological samples. This band corresponds to a second order of the CNT vibration.

Photoluminescence from SWNTs, as well as optical absorption and Raman scattering, are linearly polarized along the tube axis. This allows monitoring the SWNT orientation without direct microscopic observation. Indeed, semiconducting single-walled CNTs emit near-infrared light upon photoexcitation, described interchangeably as fluorescence or photoluminescence. However, no excitonic luminescence can be produced in metallic tubes. Their electrons can be excited, thus resulting in optical absorption, but the holes are immediately filled by other electrons of the many available in the metal. Therefore, no excitons are produced. Hence, photoluminescence is used for characterization purposes to measure the quantities of semiconducting nanotube species in a sample.

Another attractive property of these carbon nanostructures is their exceptional photothermal response. Photothermal therapy is used to reduce the size of tumors or even to eliminate them. SWNTs have been shown to serve as photothermal therapeutic agents to destroy cancer cells, using NIR laser irradiation to generate heat, following SWNTs internalization (Kam et al., 2005; Chakravarty et al., 2008).

CNTs may further be filled with different ferromagnets, therapeutics, sensors, or magnetic resonance contrast agents for applications such as sensoring (Klingeler et al., 2008), hyperthermia cancer treatment (Singh and Torti, 2013), drug delivery (Hampel et al., 2008), biosensing (Oh et al., 2013), or magnetic resonance imaging (MRI) (Sitharaman et al., 2005).

**Figure 2:**
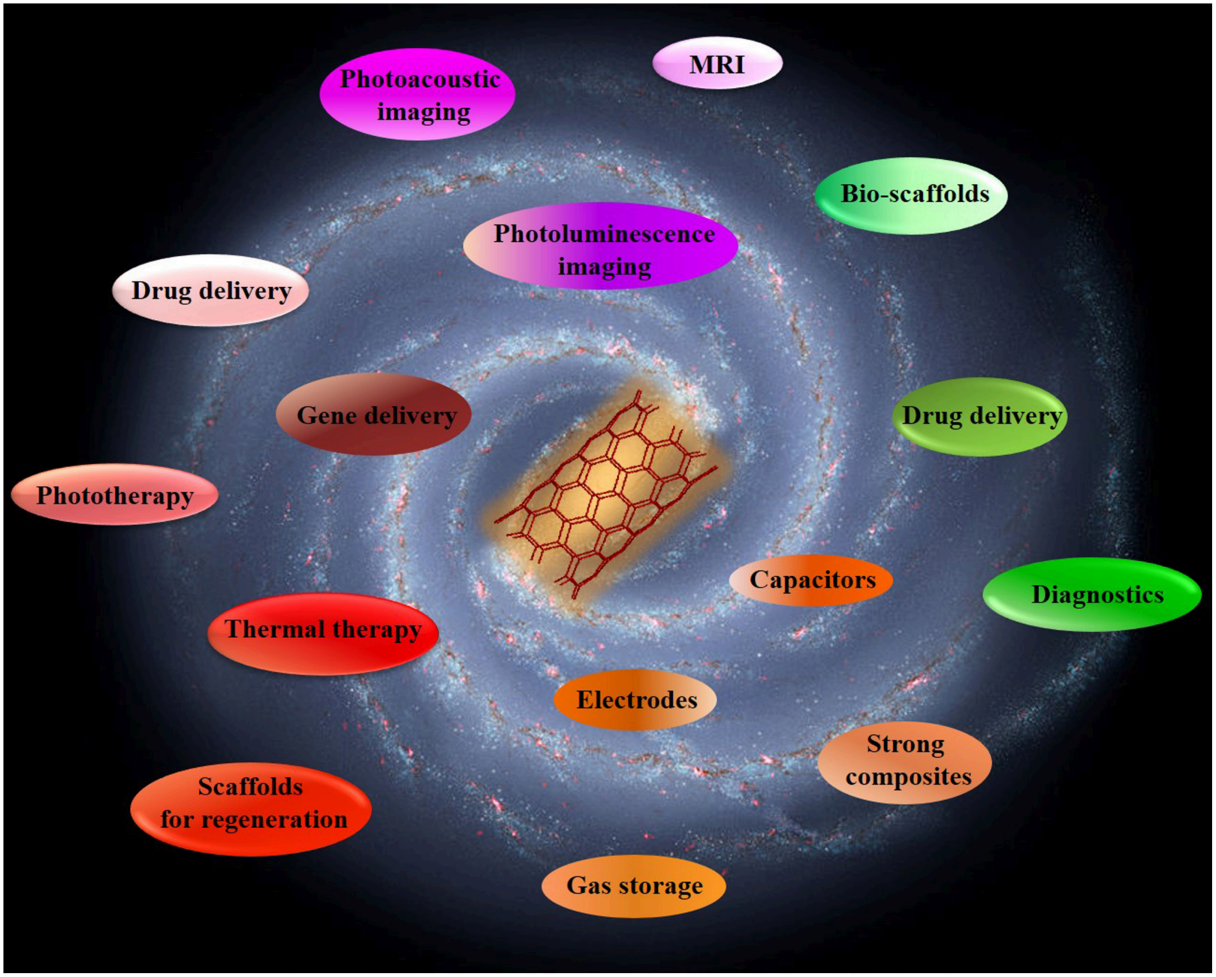
**Applications of carbon nanotubes**.

Last but not least, the high optical absorption of SWNTs can also be used in photoacoustic imaging. This method has higher spatial resolution than traditional ultrasound and deeper tissue penetration than fluorescence imaging (Xu and Wang, 2006). In 2010, a novel photoacoustic contrast agent based on Indocyanine Green dye-enhanced single-walled carbon nanotubes (SWNT-ICG) was developed which yielded a 300 fold greater photoacoustic contrast in living tissues than previously reported SWNTs with subnanomolar sensitivity (Zerda et al., 2010). Hence, thanks to the combination of their unprecedented properties, CNTs are extremely well suited for a wide variety of challenging biomedical applications (Figure 2).

### Functionalization

#### Exohedral functionalization

One of the major issues with CNTs for applications in the biomedical field is the inherent difficulty to handle them. Indeed CNTs tend to aggregate into bundles through strong attractive interactions, which are very difficult to disrupt. As grown, pristine buckytubes have highly hydrophobic surfaces and are not soluble in water or any common solvents. Introduction of functional groups onto the surface of CNTs therefore helps to solubilize them and facilitates their study (O'connell et al., 2001; Tasis et al., 2003).

Surface functionalization of CNTs may be non-covalent or covalent.

##### Non-covalent functionalization of CNTs

**Figure 3:**
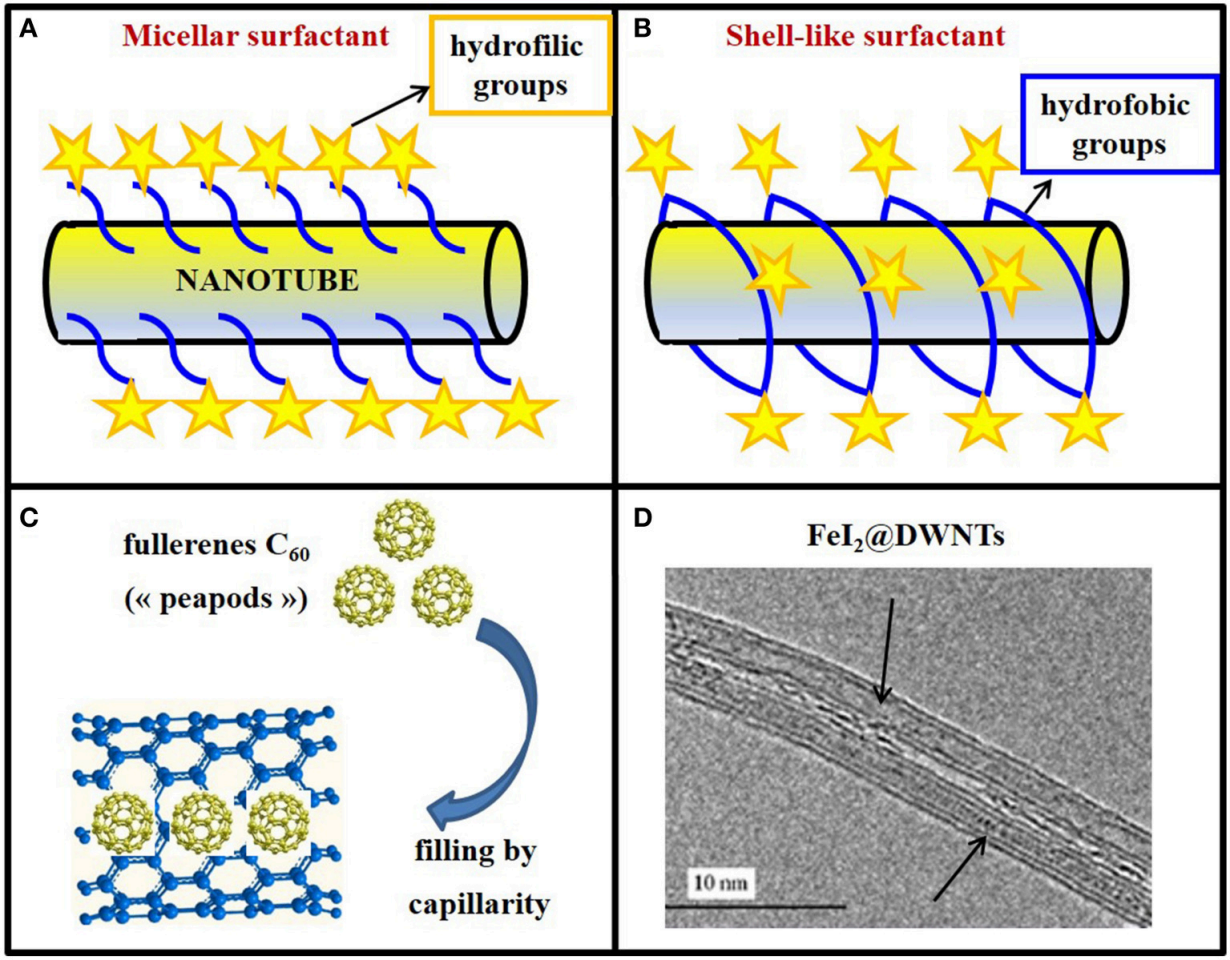
**Exohedral and endohedral functionalization of CNTs. (A)** Non-covalent surface functionalization of a buckytubes with a micellar surfactant or **(B)** with a shell-like surfactant wrapping around the nanotubes. In both cases, hydrophobic groups are evidenced by blue lines and hydrophilic ones by yellow stars. **(C)** Model of nanotubes filled with “peapods” (C_60_). **(D)** DWNTs filled on all the length with molten FeI_2_ (FeI_2_@DWNTs).

Non-covalent functionalization has many advantages, mainly the preservation of the structural and electrical properties of CNTs. Furthermore, these procedures are usually quite simple and fast, involving steps such as ultrasonication, centrifugation, and filtration. The structures that the surfactant molecules (commonly used for non-covalent functionalization) adopt when they are attached to the carbon nanotube's wall are diverse (Figure 3). In the case of charged surfactants, the dispersion of nanotubes is stabilized by electrostatic repulsion between micelles (Figure 3A); in the case of charge-neutral surfactants, the hydrophilic moieties of surfactants create a shell-like structure, which appears assembled around the nanotube (Figure 3B).

Although surfactants are efficient in the solubilization of CNTs, they are known to permeabilize the plasma membrane. Being for the greater part cytotoxic, surfactants may therefore limit biological application of such functionalized CNTs. Recently, Holzinger et al. developed an original method for the synthesis of multivalent biosensors based on non-covalent triple functionalization of SWNTs (Holzinger et al., 2010). In this study, three different pyrene derivatives were simultaneously immobilized on the nanotube's surface by π-stacking in a one-step reaction by simple dip coating of nanotubes coating electrodes.

Furthermore, single-stranded DNA molecules have been widely used to solubilize SWNTs by virtue of the π–π stacking between hydrophobic DNA base units and the nanotube's surface (Tu and Zheng, 2008). However, the main disadvantage of non-covalent functionalization when it is used for gene delivery is that DNA binding to the nanotubes may be unstable (Kostarelos et al., 2007).

In fact, an ideal non-covalent functionalization coating on CNTs for biological applications should be (1) biocompatible and non-toxic, (2) stable enough to avoid detachment from the nanotube's surface in biological solutions, especially in serum having high salt and protein contents, and having very low critical micellar concentration values, so that it remains stable after removal of excess coating molecules from the CNT suspension, (3) bear functional groups which are available for bioconjugation with antibodies or other molecules so as to enable subsequent preparation of CNT conjugates for different purposes.

Non-covalent functionalization of SWNTs by PEGylated phospholipids meets these requirements, including high water solubility of nanotubes and versatile functionalities (Liu et al., 2007, 2008). MWNTs have been grafted with polyethylenimine (PEI), a positively charged polymer, known for its excellent binding properties to DNA (Liu et al., 2005). This approach may be exploited to prepare highly sensitive and low toxic CNT-based DNA sensors (or probes) and novel safe and efficient gene delivery systems.

RNA-wrapping is another attractive method to solubilize CNTs and yielding high solubilization and no cytotoxicity. However, RNA-wrapping confers negative charges to the CNTs that make them unsuitable for DNA binding. To overcome this problem, Sanz et al. used a cationic polymer as a bridge between the negatively charged RNA wrapped around the CNT and the negatively charged plasmid DNA (Sanz et al., 2011a). Hence, the best conditions for plasmid DNA binding were obtained with PEI, but, given its cytotoxicity, the best combination for solubilization and DNA binding was poly(Lys:Phe, 1:1), which is less toxic.

##### Covalent functionalization of CNTs

Various covalent reactions have been developed to functionalize CNTs, oxidation being one of the most common. CNT oxidation is often carried out with oxidizing agents such as nitric acid (Rosca et al., 2005). During this process, carboxylic groups are formed at the ends of the tubes, as well as at defective sites on the sidewalls (Figure 3B). Despite the robustness of covalent functionalization, the intrinsic physical properties of CNTs, such as photoluminescence and Raman scattering are often modified, even destroyed after chemical reactions, due to the disrupted nanotube structure. The intensities of Raman scattering and photoluminescence of SWNTs are drastically decreased after covalent modification, reducing the potential of these materials for optical applications (Liu et al., 2009). However, a mild and green method of CNT oxidation using K_2_FeO_4_ was recently developed, producing hydrophilic CNTs with abundant surface -COOH groups (Zhang and Xu, 2015). Fe(IV) oxidation of CNTs was indeed detected at the initial defects of the carbon shell, specifically on sp^3^-C, without affecting the C=C bonds in the hexagonal rings.

Zeng et al. also observed sp^3^-carbon atoms on SWNTs after oxidation and further covalent conjugation with aminoacids (Zeng et al., 2008). However, although oxidized CNTs are rather “soluble” in water, they aggregate in the presence of salts, due to charge screening effects and thus cannot be directly used for biological applications, because of the high salt content of most biological solutions. Further modification can be achieved by attaching hydrophilic polymers, such as polyethylene glycol to oxidized CNTs, yielding CNT-polymer conjugates which are stable in biological environments (Liu et al., 2007).

Another widely used type of covalent reaction to functionalize CNTs involves cycloaddition, which occurs on the aromatic sidewalls, instead of the nanotube's ends and defects as in the case of oxidation. The 1,3-dipolar cycloaddition reactions on CNTs developed by Prato et al. is now commonly used (Pantarotto et al., 2004b).

Due to the high specific area of the CNTs, multiple copies of different molecules can be introduced onto the nanotube surface, which opens the door to perform multiple functions. For this purpose, different strategies have been reported for the double and triple covalent functionalization of buckytubes (Lamanna et al., 2011; Ménard-Moyon et al., 2011). The double covalent functionalization of CNTs based on the subsequent derivatization of oxidized CNTs by 1,3-dipolar cycloaddition and by amidation was first published in 2005 (Wu et al., 2005). CNTs were functionalized with molecules bearing primary amines blocked by orthogonal protecting groups. Hence, the use of specific conditions to selectively deprotect some of the amine functions allowed control of amine derivatization with an imaging probe and a therapeutic agent. Other double functionalization methods were based on double 1,3-cycloaddition (Pastorin et al., 2006), double amidation (Bhirde et al., 2009), a combination of 1,3-dipolar cycloaddition of azomethine ylides and arylation using diazonium salts (Brunetti et al., 2007) or a combination of amidation or arylation reactions (Stephenson et al., 2007).

Importantly, it was shown that the degree of functionalization alters tissular distribution and excretion profiles (Al-Jamal et al., 2012). Increased CNT functionalization indeed enhances renal clearance, while lower functionalization promotes reticuloendothelial system accumulation. Therefore, by tuning the degree of surface chemical functionalization of nanotubes, greater control over their organ distribution and clearance profiles *in vivo* can be achieved, which is of course essential for CNT-based diagnostics and therapeutics.

To summarize, it was proven through various studies that surface functionalized CNTs can behave in a biologically different and safer manner, compared to their pristine counterparts (Lamprecht et al., 2009; Heister et al., 2010; Marchesan et al., 2015), which may easily agglomerate into bundles and contain residual impurities, such as metal catalyst nanoparticles or amorphous carbon.

#### Endohedral functionalization

The filling of CNTs represents a striking example of matter manipulation at the nanometric scale. Indeed, the cylindrical inner cavity of CNTs can be filled with foreign materials for various applications. The study of X@CNTs (Monthioux et al., 2006) (carbon nanotubes filled with different atoms, molecules or compounds “X”) started with the incidental discovery that fullerenes could enter SWNTs, thereby forming the so-called “peapods” (Smith et al., 1998; Figure 3C).

The filling of nanotubes while they grow (*in situ* filling) can be achieved through electric arc process (Ajayan and Lijima, 1993) or CCVD procedure (Hampel et al., 2006). However, in most cases, the filling step is separated from synthesis (*ex situ* filling) and two methods can be distinguished: the filling in solution (through wet chemistry route) and the filling with a melted phase (by a physical route).

##### Filling of CNTs from solutions

This method consists in bringing into contact a concentrated solution of the desired material to be filled (generally a “precursor”) with open nanotubes. The first study on the large size MWNTs filled with metal precursors was described in 1994 (Tsang et al., 1994). Molecular dynamic simulation has shown that nucleic acids could also be inserted into the hollow cavity of CNTs in an aqueous environment, providing that the CNT diameter exceeds a critical value of 1.08 nm (Gao et al., 2003). In addition, CNTs can be filled with anticancer (Hampel et al., 2008; Tripisciano et al., 2009) or anti-malarial drugs (Sanz et al., 2011b).

In parallel, efforts have been made to fill SWNTs or DWNTs with smaller diameters. SWNTs were filled with ruthenium chloride (Sloan et al., 1998). Recently, Bortolamiol et al. described DWNTs filling with uranyl nitrate (Bortolamiol et al., 2014).

One of the biggest advantages of this method is that sensitive biological compounds with low melting point or high decomposition rate can be easily dissolved and introduced into the CNTs, which would otherwise be impossible through a physical route. However, this strategy requires CNTs to be opened, usually in aggressive conditions, which may lead to damaging of the outer wall or surface functionalization. Moreover, filling yield tends to be rather low (scarcely above 20%) and dramatically reduced with a decrease in CNT diameter, which makes the task rather difficult in the case of SWNTs and DWNTs.

##### Filling of CNTs from melted phases

The physical method involving a melted phase is more restrictive than the wet chemistry route: first, because some materials may start to decompose before they melt and second, because the melting point has to be compatible with the process.

The mechanism of the nanotube's opening during the process of filling with melted compounds is still not clearly established, but is certainly related to the chemical aggressiveness of the molten materials toward the structural defects of CNTs, mainly at the tips.

Filling yields are greater than for filling in solution, although the exact percentage of filled tubes in the entire sample is not always easily to determine, and the yield is often deduced from transmission electron microscopy (TEM) observations. In 2009, the first methodology for the quantitative assessment of the amount of material encapsulated in filled CNTs was reported, based on thermogravimetric analyses in air of the filled buckytubes (Ballesteros et al., 2009).

CNTs can be filled with melted metals (Saito and Yoshikawa, 1993), salts (Kitaura et al., 2008), semiconductors (Chimowa et al., 2011), metallic precursors for hyperthermia cancer treatment (Tîlmaciu et al., 2009), or with contrast agents for MRI (Law et al., 2014). Hence, buckytubes constitute smart carrier systems which may be filled with tailored materials to address specific demands (Figure 3D).

To conclude, the melted phase method is the first preferred method in terms of filling yields with various compounds, although its main limitation is the thermal stability of the filling material.

#### Cellular internalization and biodistribution

**Figure 4:**
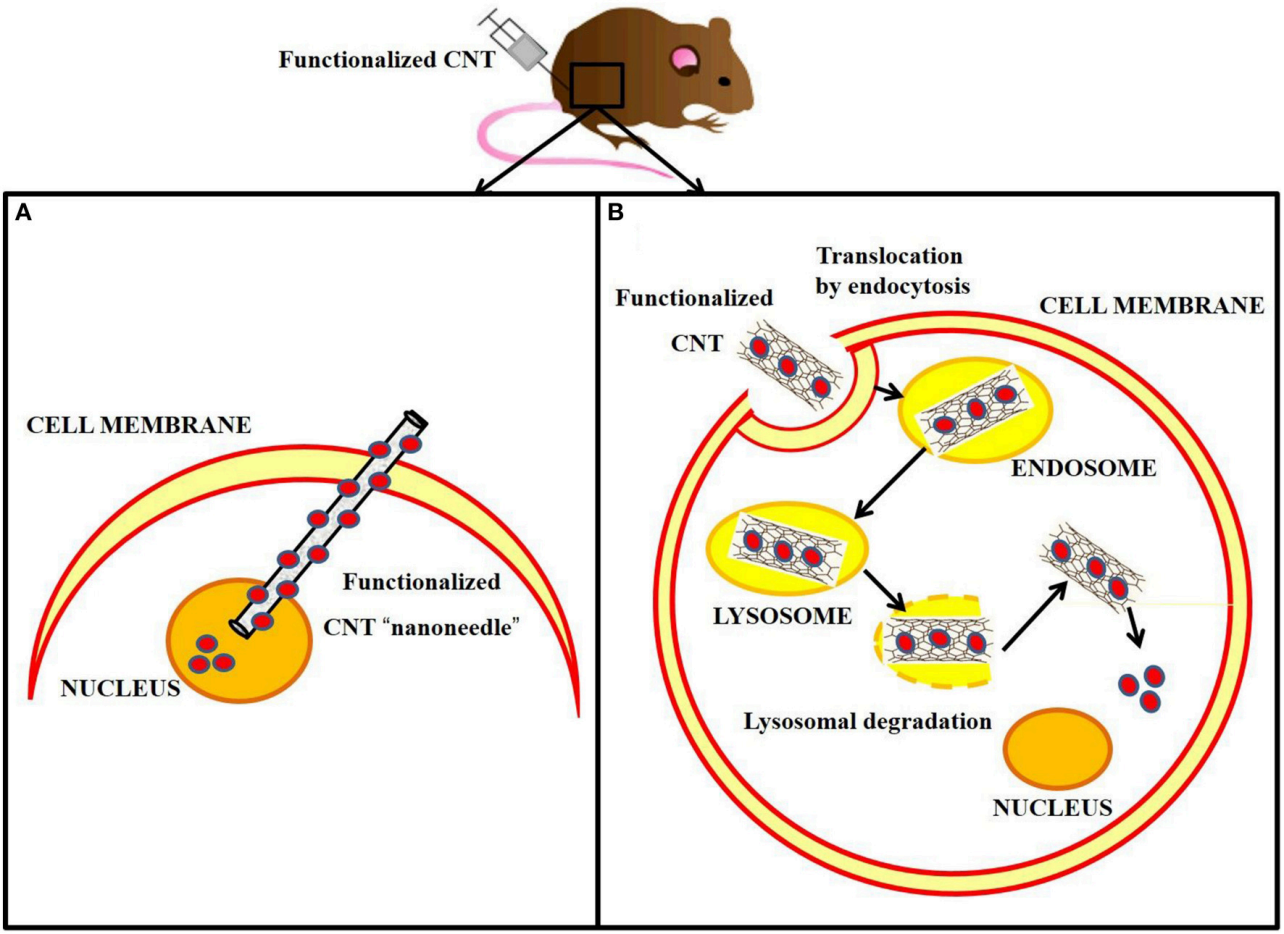
**Cellular internalization of carbon nanotubes via “nanoneedle” mechanism *vs*. endocytotic pathway, following *in vivo* injection. (A)** Functionalized CNT rapidly penetrates the cell membrane directly to the nucleus, where it releases the cargo (red circles). **(B)** Functionalized CNT is internalized in the cell by endocytosis and delivered to the endosome, which matures to a lysosome. The accumulation into the lysosome causes swelling and rupture of the vesicle followed by the release of the functionalized CNT into the cytoplasm. The cargo is then able to diffuse through the cytoplasm.

Biological barriers exist to protect the living cells from invasion of foreign nanomaterials. The biodistribution of any exogenous biomolecule is therefore primarily ruled by its ability to cross the cell membrane and gain access into the subcellular media. Pantarotto and colleagues published the first evidence that buckytubes translocate across cell membranes, describing this process as endocytosis-independent (Pantarotto et al., 2004b). Thus, it was proposed that water-soluble amino-functionalized SWNTs behave like cell-penetrating peptides and related synthetic oligomers. It was further shown that uptake of DNA by CNTs enabled gene expression (Pantarotto et al., 2004a). It was hypothesized that cationic amino-functional groups bind the nanotubes to the cell membrane, facilitating a spontaneous insertion mechanism through the biomembrane. This “nanoneedle” mechanism (Figure 4A) was thus proposed for the interaction of SWNTs with human cervical cancer cells (HeLa), when the nanotubes were observed crossing the plasma membrane barrier by TEM. Additionally, various types of functionalized CNTs were found to be taken up by a wide range of cells, some of which are deficient in phagocytotic function (fibroblasts) or lack the capacity to undergo endocytosis (fungi, yeast and bacteria) (Kostarelos et al., 2007). Hence, the term “nanosyringe” was adopted by Lopez et al. to describe a model whereby nanotubes interact with lipid bilayers by direct diffusion through the membrane (Lopez et al., 2004).

In contrast, Kam and collaborators reported the endocytotic uptake of SWNTs and SWNTs-streptavidin by mammalian cells (Kam et al., 2004; Figure 4B). Further studies showed that SWNTs functionalized with proteins and nucleic acids penetrated cell membranes by clathrin-mediated endocytosis (Kam et al., 2006).

Besides, it was shown that DNA-wrapped SWNT uptake is length-selective (Becker et al., 2007). Additionally, the functionalized-CNTs diameter appears to be an important factor since large-diameter CNTs were found to be more cytotoxic than small-diameter CNTs. Nevertheless, MWNTs allow for prolonged release of the encapsulated drug, thereby increasing its anticancer efficacy (Muzi et al., 2015).

CNT uptake was further observed by atomic force microscopy (AFM) to visualize non-covalently functionalized SWNTs and DWNTs immobilized on plasma membranes or nuclear envelopes (Lamprecht et al., 2009). Recently, the same team demonstrated folic acid mediated CNT binding to human carcinoma cells and their transport into the cytosol by AFM and molecular recognition force spectroscopy (MRFS) (Lamprecht et al., 2014).

Jin and co-workers reported the first example of exocytosis and showed that the rate of exocytosis closely matches that of endocytosis by single particle tracking (Jin et al., 2008). Neves et al. further described release of RNA-wrapped oxidized DWNTs from PC3 cells (Neves et al., 2010). CNT uptake and release occurred within 24 h, with no significant changes in cell structure or loss of viability. Cells gradually released CNTs, with a marked four-fold decrease in cellular accumulation after 12 h incubation, the presence of CNTs being negligible after 24 h. Moreover, the potential of DNA-SWNTs as long-term cellular biomarkers and sensors was also assessed (Heller et al., 2005).

Multiple studies have been performed to investigate the internalization of soluble CNTs in neurons (Cellot et al., 2010; Al-Jamal et al., 2011) and the fate of functionalized CNTs into the brain was evaluated (Bardi et al., 2013). Histological examination of sequential coronal sections after injection of functionalized MWNT-NH${}_{3}^{+}$ with lengths between 0.5 and 1 μm in the murine cortex revealed that pristine MWNT-NH${}_{3}^{+}$ were more widely dispersed in brain parenchyma compared to oxidized MWNT-NH${}_{3}^{+}$. In order to better visualize the localization of functionalized MWNT-NH${}_{3}^{+}$, TEM analysis was also performed and showed that both CNTs were predominantly uptaken by microglia neural tissue cells. Once internalized, MWNT-NH${}_{3}^{+}$ were visualized either as free individualized nanotubes in the cytoplasm or within vesicles.

Interestingly, studies of functionalized MWNT trafficking and subcellular localization in *Catharanthus roseus* plant protoplasts revealed an endosome-escape uptake mechanism (Serag et al., 2011). Moreover, at short diameters (< 100 nm), the CNTs were found to target specific cellular structures including the nucleus, plastids and vacuoles.

In conclusion, although much progress has been made toward our understanding of how nanotubes interact with cell membranes and penetrate into cells, the mechanisms of uptake, intracellular localization and biodistribution appear to vary with functionalization, length, diameter, number of walls, and concentration of CNTs. Irrespective, CNTs have emerged as promising nanocarriers for drug delivery, diagnostics, and molecular imaging.

#### Toxicity and biocompatibility

Safety is the first requirement of any material used for biomedical purposes. Together with the growing number of CNT applications in nanomedicine (Vardharajula et al., 2012; Mundra et al., 2014), questions are raised about the potential toxicity of these nanomaterials.

Toxicity can be assessed through exposure of cultured cells to suspensions of CNTs, prepared with or without addition of surfactant, and dispersion by sonication. Indeed surfactants, present in excess in CNT suspensions, are known to be highly toxic for cells and may therefore contribute to the observed toxicity of CNT samples whenever present (Dong et al., 2008). The metal catalyst content in CNTs can generate free radicals that can cause oxidative damage to cells and membranes, if they are not removed during purification (Plata et al., 2008). This is especially important since the annual production of buckytubes is now reaching hundreds of tons per year.

To address the possible side effects of CNTs on human health and environment, the toxicology of CNTs has been investigated on animal models. In a pilot study, Poland et al. noticed the structural similarity of CNTs with asbestos fibers. Following intraperitoneal injection, the mesothelial lining of the mouse body cavity was exposed to large MWNTs (length 10–50 μm, diameter 80–160 nm), which were simply sonicated in 0.5% bovine serum albumin solution, without any surface functionalization (Poland et al., 2008). Hence, this study cannot be related to functionalized CNTs with biocompatible coatings recommended for biomedical applications. Moreover, it is worth noting that functionalized SWNTs used routinely in biomedical research have a length of 50–300 nm and a diameter of 1–2 nm, which is much smaller than the MWNTs used by Poland et al. (Yang et al., 2008). Other studies on unpurified, pristine CNTs have reported that buckytubes can induce granuloma, fibrosis, or inflammation following lung administration (Muller et al., 2005; Shvedova et al., 2005). To determine whether biodegradation might render nanotubes non-inflammatory, *in vivo* tests on mice were performed, showing that non-degraded nanotubes induced the formation of tissue granulomas, whereas no granulomas were observed in the lungs of mice exposed to biodegraded SWNTs (Kagan et al., 2010). Moreover, carbon nanostructures are susceptible of inducing formation of reactive oxygen species (ROS). Yang et al. studied carbon nanohorns, a particular form of carbon, also discovered by Iijima et al. (1999) and showed that an excessive uptake by lysosomes induced lysosomal dysfunction and consequent generation of ROS in the mitochondria (Yang et al., 2014).

However, more recent studies have revealed that CNTs can be made biocompatible through various dispersion and functionalization strategies. Hence, Sayes et al. reported that the toxicity of CNTs was dependent on the density of surface functional groups, with minimal toxicity for heavily decorated tubes (Sayes et al., 2006). Additionally, hydrophilic groups introduced onto the CNTs surface render them more biocompatible and biodegradable (Bianco et al., 2011). Besides, structural defects on the SWNT surface allow oxidative enzymes to degrade the buckytubes under environmentally relevant settings (Allen et al., 2008). Simmons and co-workers exploited a non-covalent strategy to attach carboxylic functional groups through π–π stacking interactions, thereby creating stable aqueous dispersions and limiting cytotoxicity (Simmons et al., 2009). RNA-wrapping is another attractive method to solubilize CNTs without any toxic effect (Jeynes et al., 2006). Moreover, Yang et al. showed that covalently PEGylated SWNTs exhibited an ultralong blood circulation half-life in mice and no acute toxicity has been reported even at a high dose (24 mg kg^−1^) (Yang et al., 2008). Recently, Maruyama and co-workers prepared biocompatible MWNTs that were endocytosed mainly through clathrins by human normal bronchial epithelial cells and mesothelium cells, without any appararent toxicity (Maruyama et al., 2015).

Hence, it appears that pristine CNTs and CNTs without serum-stable functionalization show toxicity to cells at moderate concentration, while serum-stable, functionalized CNTs show minor toxicity even at high doses.

## Development of biosensors over time

The first biosensors appeared with the development of electrochemical devices for detection of analytes in the 1950s. The first and most famous of these is the electrochemical oxygen biosensor described by Leland Clark Jr in 1956 (Clark oxygen electrode), consisting of a platinum cathode at which oxygen is reduced and a silver/silver chloride reference electrode (Clark, 1956). Clark and Lyons later combined this electrode with glucose oxidase incorporated in a dialysis membrane to measure the concentration of glucose in solution (Clark and Lyons, 1962). A couple of years later the first “enzyme electrode” was described by Updike and Hicks in 1967 to quantify glucose in solution and in tissues *in vitro*, engineered through immobilization of glucose oxidase in a polymerized gelatinous membrane that coated a polarographic oxygen electrode (Updike and Hicks, 1967), thereby serving as an enzyme transducer to catalyze an electrochemical reaction upon recognition of glucose (Clark and Lyons, 1962).

In 1969 the first potentiometric enzyme electrode was developed by Guilbault and Montalvo, the urea sensor, based on immobilization of urease onto an ammonium-selective liquid membrane electrode (Guilbault and Montalvo, 1969).

**Figure 5:**
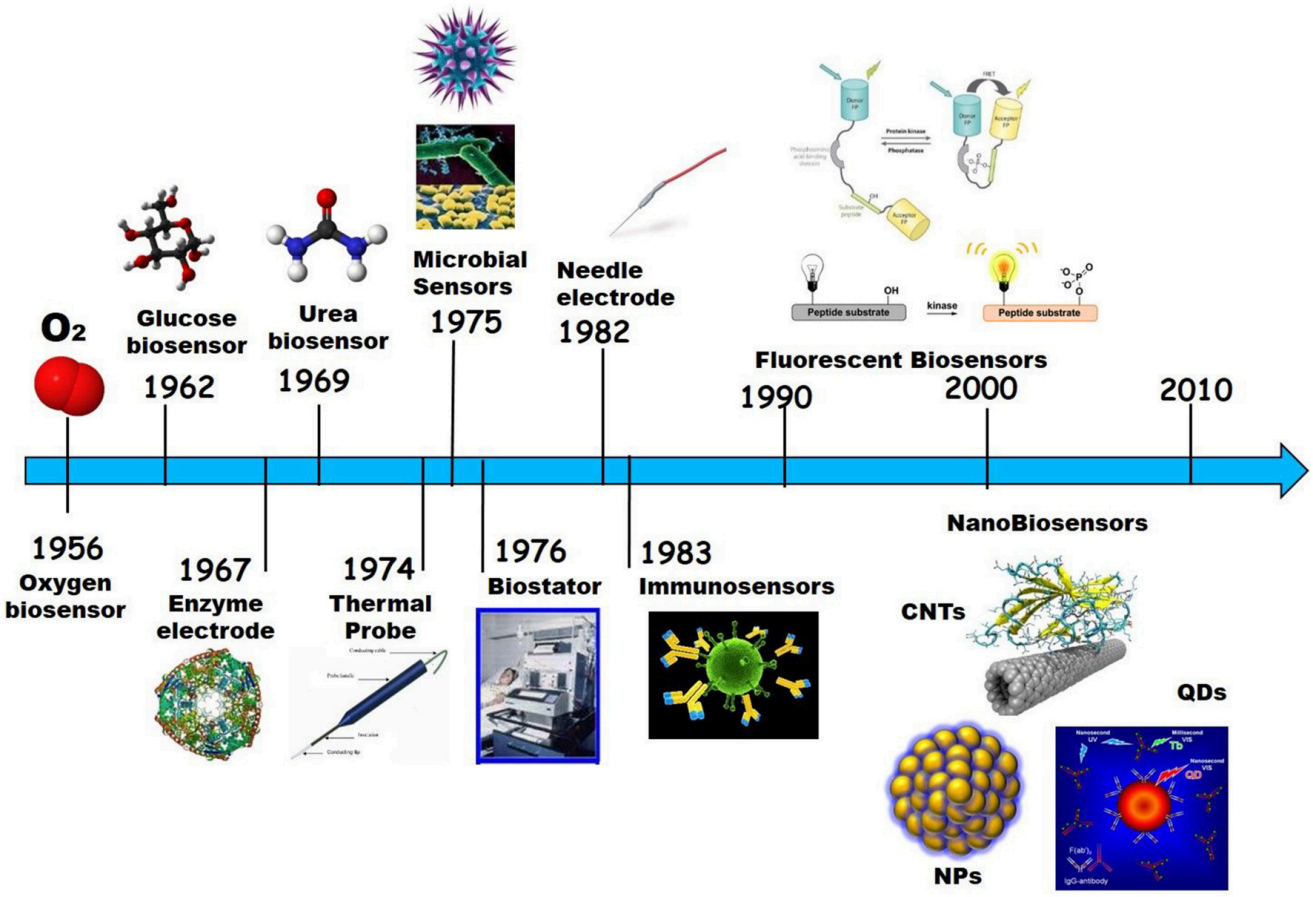
**Biosensor development timeline**.

Ever since a broad range of biosensors have been developed for *in vitro* and *in vivo* applications, whose nature varies from enzymatic, to antibody, polypeptide, aptamer, or nucleic acids-based. Likewise, the mechanism of transduction of biosensors has evolved together with demand and technologies available, from electrochemical and electronic biosensors to thermic biosensors, that measure changes in temperature associated with the amount of heat generated by an enzyme-catalyzed reaction; microbial biosensors, that integrate micro-organisms with a physical transducer, such as an electrochemical device, to monitor specific analytes or biomarkers typically through changes in respiration activity or production of electroactive metabolites; immunobiosensors based on recognition of target species by recombinant antibodies or antibody fragments; optical biosensors, based on differences in optical diffraction, changes in the emission of light signals upon recognition of their target; and more recently nanobiosensors based on nanomaterials which began to appear at the turn of the Twentieth century (Turner et al., 1987; Morris, 2010; Turner, 2013; Figure 5).

## Carbon nanotubes for biosensing applications

With the explosion of nanotechnologies and the emergence of nanomaterials with unique physicochemical properties, a new class of biosensors referred to as nanobiosensors, which includes nanoparticles and nanotubes, has been developed, that combines the advantages of nanomaterials, notably their small size and large surface/volume ratio, with the functionality of “macro”-biosensors (Ferrari, 2005; Holzinger et al., 2014).

Nanomaterials offer attractive opportunities for biosensing applications, to increase sensitivity and lower detection limits. This enhanced performance is associated with the small size of nanomaterials, which endows them with a large surface/volume ratio. This high specific area enables immobilization of greater concentrations of bioreceptor units relative to biosensor surface/volume. Moreover, the inherent physicochemical properties of many biomaterials enable them to act as transducers.

CNTs offer several assets for detection purposes. By virtue of their unprecedented structural, mechanical, electronic, and optical properties CNTs offer several features of interest to engineer new generation probes (Munzer et al., 2013; Holzinger et al., 2014; Mundra et al., 2014). First, they constitute scaffolds/platforms which may be functionalized through conjugation of several entities, thereby potentially enhancing recognition and signal transduction processes, as opposed to mono-conjugated biosensor species, but also providing means to multifunctionalize and therefore to multiplex. Through their ability to conduct electricity (approximately 100 times greater than copper wires), CNTs are well suited for transduction of electric signals generated upon recognition of a target. Their thermal conductivity is higher than diamond, and their strength approximately 100 times greater than steel. Last but not least, the ability of CNTs to cross biological membranes readily makes them applicable *in vivo* with minimal invasiveness, and they may further be employed for photoacoustic imaging.

**Figure 6:**
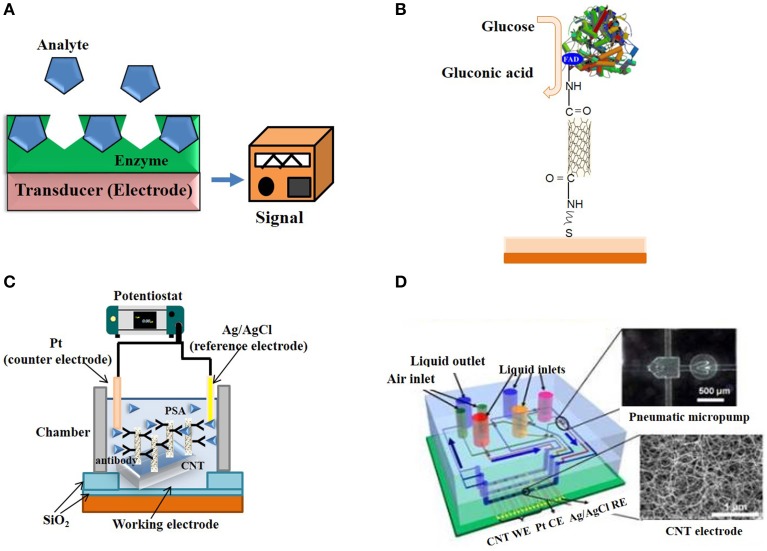
**Electrochemical and Electronic CNT biosensors. (A)** Typical design of an enzyme-based electrochemical biosensor. **(B)** SWNT electrically-contacted glucose oxidase electrode. **(C)** Schematic illustration of a label-free amperometric biosensor for PSA detection. **(D)** Schematic illustration of a microfluidic chip based on CNT electrodes (Yuichi et al., 2009).

**Figure 7:**
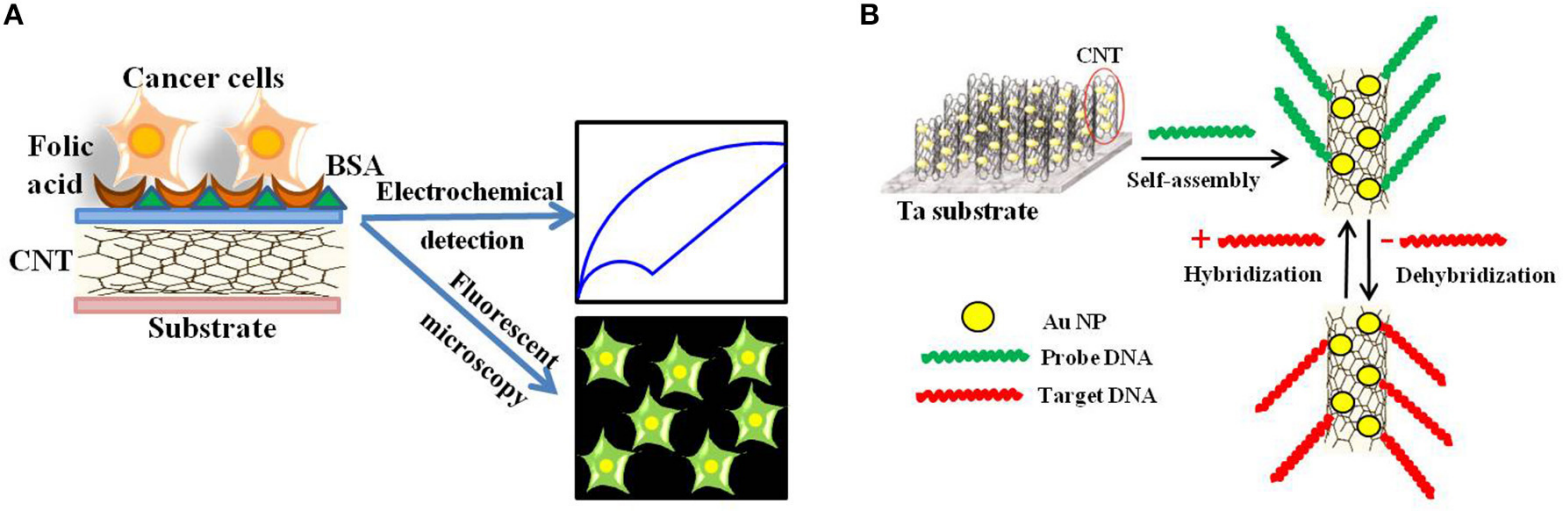
**Electrochemical and Electronic CNT biosensors for Cancer Detection. (A)** Schematic illustration of the folic acid-targeted cytosensing strategy for an enhanced electrochemical detection of cancer cells using polydopamine-coated carbon nanotubes. **(B)** Schematic representation of an electrochemical DNA biosensor for cancer detection based on gold nanoparticles/aligned CNTs.

**Figure 8:**
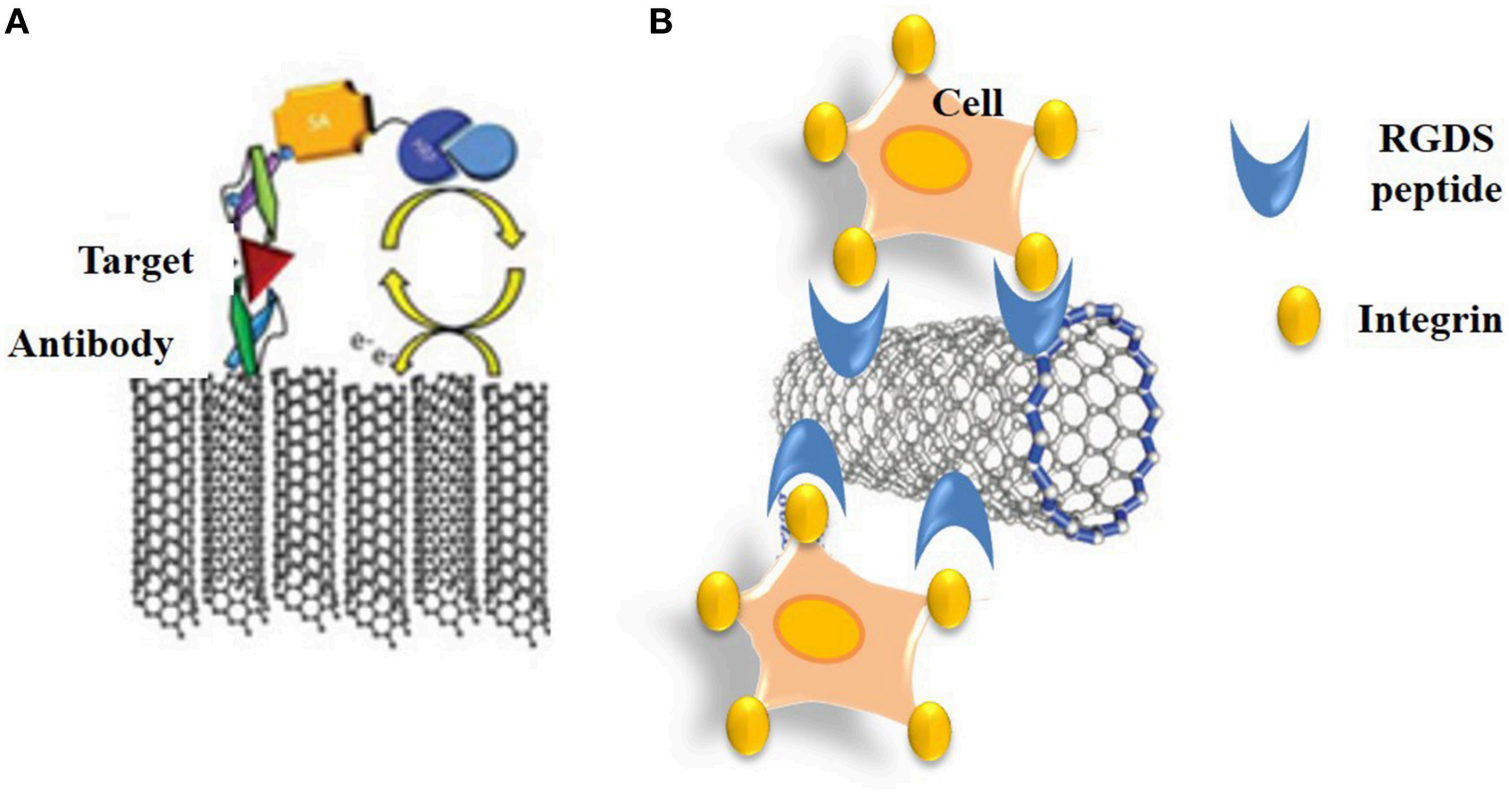
**Immuno-CNT biosensors. (A)** Schematic representation of an electrochemical CNT-immunosensor that combines a sensing interface for target detection with a sandwich-type electrochemical immunoassay for amplification of the signal. **(B)** Cytosensing immunosensor based on functionalization of SWNTs with RGDS peptides that recognize cell surface integrin receptors.

**Figure 9:**
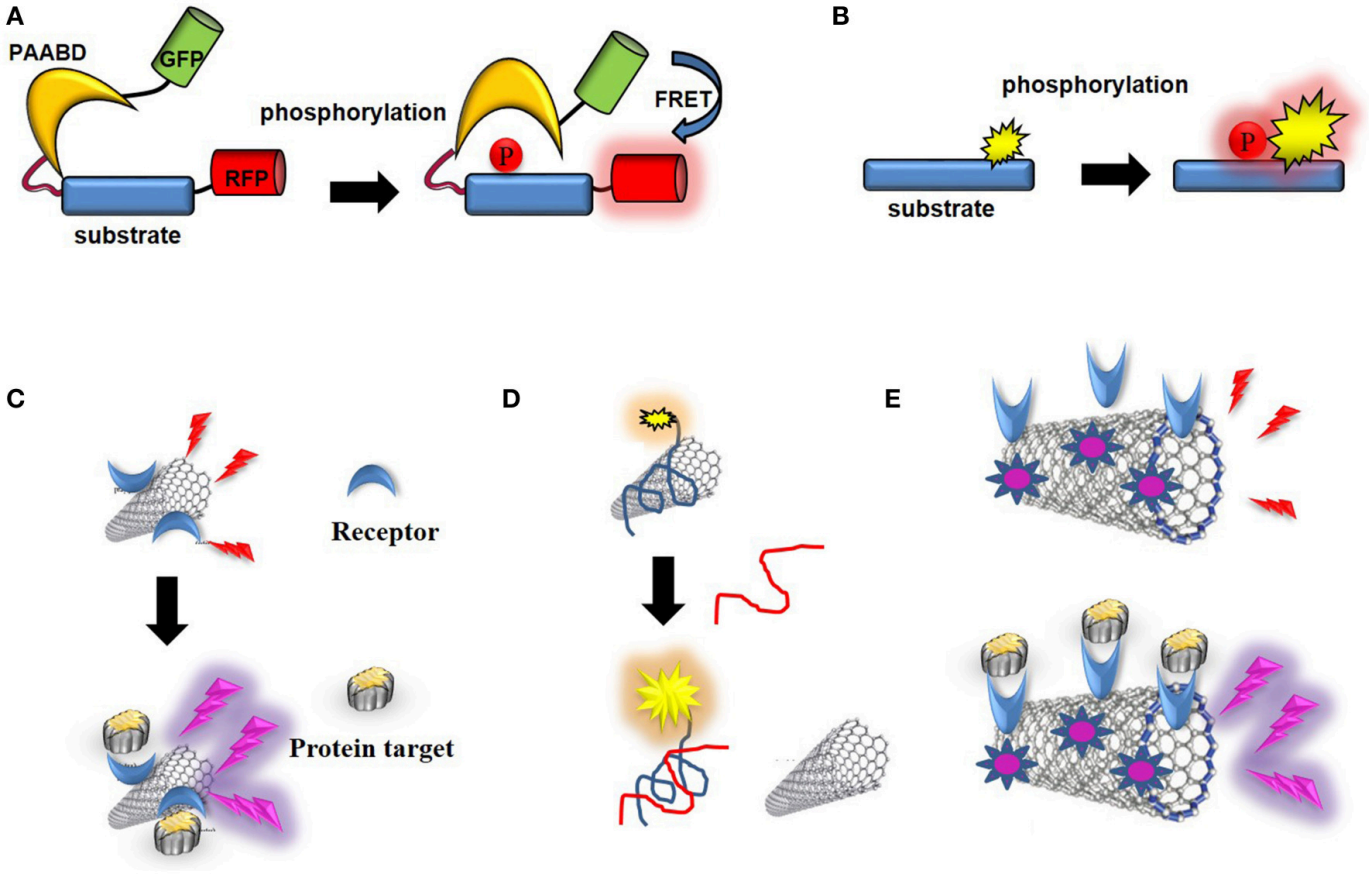
**Optical CNT biosensors. (A)** Schematic representation of a genetically-encoded FRET kinase biosensor: the donor (GFP) and acceptor (RFP) proteins are brought in close proximity which enables FRET following a phosphorylation-mediated intramolecular conformational change. **(B)** Schematic representation of non-genetic, environmentally-sensitive peptide-based biosensor: the probe environment is altered upon phosphorylation of the substrate. **(C)** CNT-biosensor based on wavelength shift of fluorescence upon target binding to a receptor conjugated into the SWNT. **(D)** Optical biosensor of ssDNA: fluorescence of a dye-labeled oligonucleotide is quenched upon non-covalent assembly with SWNT, until the ssDNA target binds and releases the labeled oligonucleotide from the SWNT. **(E)** Photoacoustic detection of integrins by indocyanine-labeled SWNT-biosensor conjugated with RGD peptides (Zerda et al., 2010).

An ever-growing number of CNT-conjugates have been developed for detection of DNA biomarkers, cell-surface sugars, protein receptors and enzymes. Depending on their mechanism of target recognition and transduction, these biosensors are broadly subdivided into electronic transducers, electrochemical CNT-biosensors, immunosensors, and optical CNT-based biosensors (Figures 6–**9**).

Different classes of CNT biosensors have been developed to probe a wide variety of cancer biomarkers through conjugation or complexation of DNA or aptamers, antibodies, peptides, proteins, or enzymes (Ferrari, 2005; Portney and Ozkan, 2006; Choi et al., 2010). The incorporation of CNTs into biosensing devices has enabled the development of highly sensitive electrochemical biosensors for early-stage detection of biomarkers of various diseases including cancer (Wang and Dai, 2015). CNT-based immunosensors are still in the nascent stage and there are many challenges to overcome for the successful commercialization of the concepts (Veetil and Ye, 2007). More recent developments have led to application of CNT-biosensors as imaging probes, in particular for photoacoustic imaging.

### Electrochemical and electronic CNT biosensors

The larger part of biosensors developed to date are electrochemical. They are popular due to their low-cost, relatively fast response times, ease of use, and small size. Enzyme-coupled electrochemical biosensors are based on enzymatic catalysis of a reaction that produces electro-active species, thereby generating a measurable electric signal. The biosensor generally contains a reference electrode, a working electrode and a counter electrode. The target analyte is recognized by enzymes immobilized on the working electrode, catalytic activity of which may cause either electron transfer, thereby producing a current or contributing to produce a voltage (Figure 6A). Enzymes are optimal biorecognition molecules, because they provide excellent selectivity for their targeted substrate and have high catalytic activity.

CNTs have been recognized very promising materials for enhancing electron transfer, thanks to their electrical and electrochemical properties, which make them suitable for integration into electrochemical biosensors (Balasubramanian and Burghard, 2006; Tothill, 2009; Bohunicky and Mousa, 2011; Holzinger et al., 2014; Kumar et al., 2015; Wang and Dai, 2015). Their small size, large surface area, high conductivity, high chemical stability and sensitivity (Zhao et al., 2002), high electrocatalytic effect, and fast electron-transfer rate (Lin et al., 2004) make them extremely well suited for biosensing applications relying on enzymatic reactions and/or that generate electro-active species. A wide variety of electrochemical CNT-biosensors have been developed to detect ions, metabolites and protein biomarkers (Wang and Dai, 2015). For instance, several CNT-glucose biosensor based on conjugation of glucose oxidase have been engineered (Lin et al., 2004; Patolsky et al., 2004). Patolsky et al. reported on the structural alignment of glucose oxidase (GOx) on electrodes using SWNTs as electrical connectors between the enzyme redox centers and the electrode (Figure 6B). They demonstrated that the surface-assembled GOx was electrically contacted to the electrode by means of the SWNTs, which acted as conductive nanoneedles that electrically wire the enzyme redox-active site to the transducer surface. Cholesterol biosensors consisting of modified screen printed electrodes with cholesterol esterase, peroxidase, oxidase and MWNTs yield highly sensitive means of quantifying total cholesterol in blood (Li et al., 2005).

Electrochemical biosensors based on functionalized CNTs have further been developed for detection of nitric oxide (Santos et al., 2013), epinephrine sensing (Prasad et al., 2013), and dopamine monitoring in rat striatum (Kress et al., 2014). An RNA aptasensor for detection of disease-related glycoproteins in blood was developed by coating SWNTs grafted with protein-specific RNA aptamers on an alumina electrode (Zelada-Guillén et al., 2013).

Zhang et al. reported the selective detection of cellular nitric oxide (NO) by single-stranded d(AT)15 DNA oligonucleotide adsorbed and wrapped around SWNTs (near-infrared fluorescent single-walled carbon nanotubes) (Zhang et al., 2011). Jin et al. developed SWNT-based biosensors of H_2_O_2_ which were applied to single molecule imaging in human epidermal carcinoma cells (Jin et al., 2010).

Electronic monitoring of electrochemical biosensors can be distinguished by the mechanism of transduction as amperometric, potentiometric, conductometric, voltametric, or piezoelectric systems.

Potentiometric biosensors measure the oxido-reduction potential of an electrochemical reaction, and are therefore based on biological reactions that produce or absorb hydrogen ions, causing a net change in pH, which can be measured as an electrical signal, potential, at the surface of a pH-meter probe. Potentiometric biosensors make use of ion-selective electrodes in order to transduce the biological reaction into an electric signal.

Amperometric biosensors sense a current produced when a potential is applied between two electrodes and can therefore detect electro-active species present in biological samples, which are most often produced thanks to enzymes.

Piezo-electric biosensors are based on piezo-electric crystals (such as quartz) that vibrate under the influence of an electric field. Their resonance frequency is proportional to the mass of adsorbed material and therefore changes as molecules adsorb or desorb from the surface of the crystal.

In recent years, a large variety of amperometric biosensors based on CNT-modified electrodes have been engineered. Fei and co-workers carried out detection of cysteine on Pt/CNT electrodes by cyclic voltammetry (Fei et al., 2005). Antiochia et al. reported an amperometric CNT-biosensor developed by coating CNT with a polymer of dihydroxybenzaldehyde (Antiochia et al., 2004). Moreover, CNT-arrayed electrodes coated with anti-PSA antibodies placed within a chamber were used to sense PSA against a Pt wire counter electrode (Figure 6C; Okuno et al., 2007). Biosensors based on CNTs arrayed on a chip, in combination with pneumatic micropumps have been engineered and applied to the simultaneous detection of several biomarkers (PSA-mAb and human chorionic gonadotropin hCG antibodies) (Figure 6D; Yuichi et al., 2009).

#### Electrochemical and electronic CNT biosensors for cancer detection

Feng et al. reported a disposable paper-based bipolar electrode (BPE) for the sensitive electrochemiluminescent detection of prostate specific antigen (PSA) and showed that its response was significantly improved after modification of the BPE cathode with MWNTs (Feng et al., 2014). Besides, electrodeposition of CNTs and their subsequent functionalization with proper enzymes is used to ensure sensitivity and specificity in electrochemical biosensing. Thus, MWNTs were used to develop biosensors based on microsomal cytochrome P450 to electrochemically detect drugs used in the treatment of breast cancer (Baj-Rossi et al., 2012). Deposition of the CNT-enzyme nanostructures onto the electrodes lowered the limit of drug detection to fit the therapeutic range even in human serum. Additionally, the same team synthesized a multi-array sensor platform by electrodeposition of chitosan/MWNTs to detect several endogenous metabolites (glucose, lactate) and drugs (etoposide, mitoxantrone and etodolac) simultaneously, whilst also monitoring pH and temperature for biosensing calibration (Baj-Rossi et al., 2014).

A screen-printed carbon electrode used as the signal transducer of a dsDNA-based biosensor was modified by MWNTs and colloidal gold nanoparticles (GNPs) for testing berberine, an isoquinoline plant alkaloid with significant antimicrobial and anticancer activity (Ovádeková et al., 2006).

Detection of volatile organic compounds (VOCs) in human breath to diagnose lung cancer is becoming an important method for widespread screening, due to its facility and low cost advantages. Thus, tricosane (C_23_H_48_)-functionalized SWNTs biosensor showed pronounced sensitivity toward polar VOC molecules, which can donate electrons to the nanotubes after being absorbed (Liu et al., 2011).

D-(+)-galactose conjugated SWNTs were synthesized using molybdenum electrodes for application as biosensors to detect cancer marker galactin-3 (Park et al., 2011).

Later, Zheng and collaborators developed folic acid-functionalized polydopamine-coated carbon nanotubes for the electrochemical detection of HeLa and HL60 cancer cells over-expressing the folate receptor (Zheng et al., 2012; Figure 7A).

Fayazfar et al. reported on a new platform based on electrochemical growth of gold nanoparticles on aligned MWNTs for sensitive label-free DNA detection of the TP53 gene mutation (Figure 7B; Fayazfar et al., 2014). The electrode modified with vertically aligned MWNTs and gold nanoparticles improved the density of the DNA probe as well as the sensor sensitivity, displayed reproducibility and stability for 2 weeks, and could be conveniently regenerated via dehybridization in hot water. Alternatively, Au-Ag alloy-coated MWNTs were used as sensing interface for ultrasensitive detection of volatile biomarkers of MGC-803 gastric cancer cells (Zhang et al., 2014b).

Recently, Shobba et al. evaluated the properties of both SWNTs and MWNTs for early-stage detection of prostate cancer, through functionalization with DNA strands that detect PSA present in blood samples (Shobha and Muniraj, 2015).

CNTs have also emerged as promising sensing platforms for amperometric detection and quantification of clinically relevant metabolites such as glucose, cholesterol, lactate and glutamate, and for detection of cancer biomarkers such as alpha-fetoprotein, CEA, PSA, DNA, or microRNA biomarkers (Dey et al., 2013).

Several amperometric, impedimetric, and field-effect transistors (FET) CNT-biosensors have been developed for detection of cancer biomarkers and cells over the past couple of years. For instance, a FET-CNT immunosensor was developed for detection of osteopontin (OPN), a biomarker of prostate cancer by attaching a genetically-engineered single chain variable fragment protein with high binding affinity for OPN, and employed to monitor this biomarker in a background of concentrated bovine serum albumin (Lerner et al., 2012). A vertically aligned carbon nanotube-based impedimetric biosensor was fabricated through a photolithography process on Ni/SiO_2_/Si layers for the detection of SW48 cells, isolated from grade IV human colon tumors (Abdolahad et al., 2012, 2013).

Bareket et al. prepared a rapid, sensitive, selective and inexpensive CNT-modified screen-printed electrode to monitor the amperometric response to formaldehyde released from U251 human glioblastoma cells in response to treatment with formaldehyde-releasing anticancer prodrugs (Bareket et al., 2010).

More recently, a nanobiosensor was engineered for the detection of liver cancer cells (HepG_2_) by using real time electrical impedance sensing, through assembly of CNT multilayers and antibodies to epithelial cell adhesion molecules on an indium tin oxide electrode surface (Liu et al., 2014). Detection of tumor cell antibodies caused increase of the electron-transfer resistance and the electrochemical impedance increased in a linear fashion with the logarithm of cancer cells concentration.

### CNT immunosensors

Immunosensors rely on recognition of antigens by recombinant antibodies or antibody fragments which can be immobilized onto substrates and constitute the receptor moiety of the biosensor. In 1983, Liedberg et al. developed the first immunosensor through immobilization of antibodies onto a Chip, thereby designing the precursor of the widely used BIAcore system, which transduces immuno-recognition of analytes by surface plasmon resonance (Liedberg et al., 1983). Ever since, a wide variety of affinity reagents have become available for selective recognition of biomarkers and target analytes, ranging from recombinant antibodies and antibody fragments (scFvs and Fabs) that can be selected from phage display libraries. To date a large number of immunosensors have been engineered to probe HIV, hepatitis and other viral diseases, to test for drugs and monitor the presence of undesired or toxic compounds in the environment. Although, the larger number of these are electrochemical, both piezoelectric immunosensors based on antibody-surface coated quartz crystals, and FET immunosensors have been developed through conjugation of antibodies to conductive nanomaterials (Veetil and Ye, 2007; Kierny et al., 2012). Over the more recent years new generations of nano-immunosensors have been engineered by immobilizing recombinant antibodies or antibody fragments onto CNTs, nanowires, nanoparticles, and quantum dots, thereby enhancing binding capacity and sensitivity thresholds compared to more traditional biosensors. Electrochemical immunosensors combine a sensing interface for target detection with a sandwich-type electrochemical immunoassay for amplification of the signal (Figure 8A). Electrochemical immunosensors have also been developed for cytosensing through functionalization of SWNTs with RGDS peptides that recognize cell surface integrin receptors (Figure 8B).

#### CNT immunosensors for cancer detection

A wide variety of carbon-nanotube-based immunosensors have been developed to probe cancer biomarkers (Veetil and Ye, 2007; Kierny et al., 2012). For instance, Rusling and co-workers developed an electrochemical immunosensor based on SWNT forests for the attachment of the enzymes or antibodies by amidation (Rusling et al., 2009). Squamous cell carcinomas of head and neck were detected thanks to an ultrasensitive electrochemical immunosensor based on SWNT forests incorporating antibodies to Interleukin-6 (Il-6) and horseradish peroxidase enabling detection of very low and elevated levels of Il-6 (Malhotra et al., 2010). A multiplexing electrochemical immunosensor based on screen-printed carbon electrodes was developed for simultaneous detection of PSA and Interleukin-8 (Il-8) (Wan et al., 2011). Recently, an impedimetric immunosensor of human epidermal growth factor receptor 2 (HER2) was developed by modification of a gold nanoparticle-decorated MWNT-ionic liquid electrode (Arkan et al., 2015). Gold nanoparticles were used to enhance the extent of immobilization and to retain the immunoactivity of the HER2 antibody Herceptin on the electrode. This biosensor enabled detection of low concentrations of HER2 in serum samples of breast cancer patients and exhibited a linear increase in charge transfer resistance with the concentration of HER2.

### Optical CNT biosensors

By definition, optical biosensors report on detection of target biomolecules or analytes through changes in the emission of light (UV, visible, or infrared) (Figure 9A).

Following the first fiber optic biosensor or “optode” described by Lubbers and Oppitz to measure carbon dioxide, oxygen and alcohol, respectively (Lubbers and Opitz, 1975; Völkl et al., 1980), a wide variety of optical biosensors have been developed to study and report on dynamic biomolecular processes *in vitro*, in living cells and *in vivo* (Cooper, 2002; Martins et al., 2013). Optical biosensors can be distinguished according to the method used to readout target detection (surface plasmon resonance, absorbance, reflectance, fluorescence, phosphorescence, luminescence, wavelength intensity, lifetime, anisotropy, quenching, and fluorescence energy transfer) or by their mechanism of recognition (probing biosensors and reacting biosensors). Probing biosensors monitor differences in the interaction/affinity between analyte and recognition domain of the sensor which lead to changes in optical response. Reacting biosensors exhibit different optical responses related to chemical processes (chemisorption, catalytic reaction, formation of new chemical bonds, etc.). Over the past decade, a number of optical biosensors based on surface plasmon resonance, waveguides, and resonant mirrors have been developed to monitor label-free targets *in vitro* (Cooper, 2002). Colorimetric biosensors measure changes in light absorption as reactants are converted to products. Photometric light intensity biosensors detect changes in fluorescent or bioluminescent processes associated with changes in the spectral properties of probes involved directly or indirectly in target detection.

Fluorescence offers particularly attractive advantages for biosensing applications, such as its high inherent sensitivity and the opportunity to image dynamic processes in living cells by fluorescence microscopy in a non-invasive fashion with high spatial and temporal resolution (Zhong, 2009). Target recognition is transduced through emission of a fluorescent signal which differs from that of the biosensor in its unbound state. Fluorescent biosensors have been designed to recognize and report on the presence, activity or conformation of a given target in a specific and quantitative fashion, thereby providing means to probe its dynamic molecular behavior through sensitive changes in fluorescence.

The first fluorescent biosensors were developed in the 1990s (de Silva et al., 1997; Johnson, 1998; Terai and Nagano, 2008). Although they were initially based on incorporation of synthetic probes that are sensitive to changes in their environment into biosensor receptor moieties, the discovery of the Green Fluorescent Protein (GFP) and its engineering into genetically-encoded reporters paved the way for development of genetically-encoded fluorescent biosensors (Tsien, 1998, 2005; Ellenberg et al., 1999; Bastiaens and Pepperkok, 2000; Lippincott-Schwartz et al., 2001; Wouters et al., 2001; Zhang et al., 2002; Lippincott-Schwartz and Patterson, 2003; Shaner et al., 2005, 2007; Giepmans et al., 2006; Fernandez-Suarez and Ting, 2008). This class of biosensors relies on ectopic expression of genetically-encoded autofluorescent protein (AFP) fusions with receptor domains that recognize the target of interest in living cells. For the larger part these are single-chain biosensors which respond to enzymatic activities through fluorescence resonance energy transfer (FRET) (Ibraheem and Campbell, 2010; Morris, 2010; Van and Morris, 2013). For instance genetically-encoded FRET biosensors of protein kinases, also known as KARs (kinase activity reporters) incorporate a pair of genetically-encoded AFPs together with an enzyme-specific substrate sequence and a phosphoaminoacid binding domain (PAABD). When the substrate sequence is phosphorylated by the kinase of interest, it preferentially interacts with the PAABD, thereby inducing an intramolecular change which brings the AFPs closer and promotes fluorescence energy transfer between the donor and the acceptor (Tsien, 1998; Zhang et al., 2002; VanEngelenburg and Palmer, 2008; Aye-Han et al., 2009; Wang et al., 2009; Ibraheem and Campbell, 2010; Morris, 2010; Figure 9A).

More recent efforts made by chemists have yielded a palette of environmentally-sensitive synthetic fluorophores that respond to changes in the polarity of their environment, becoming more fluorescent in non-polar solvents or upon interaction with a hydrophobic target protein with enhanced spectral properties for *in vivo* imaging which can be conjugated to peptide/protein scaffolds, yielding attractive alternatives to their genetically-encoded counterparts (Tsien, 1998, 2005; Ellenberg et al., 1999; Bastiaens and Pepperkok, 2000; Lippincott-Schwartz et al., 2001; Wouters et al., 2001; Zhang et al., 2002; Lippincott-Schwartz and Patterson, 2003; Shaner et al., 2005, 2007; Giepmans et al., 2006; Fernandez-Suarez and Ting, 2008; Lavis and Raines, 2008; Loving et al., 2009). These systems are more readily applicable *in vitro* and can further be microinjected or introduced into living cells through facilitated delivery. They have proven sensitive and particularly well suited to monitor protein kinase activities (Pazos et al., 2009; Wang et al., 2009; Morris, 2010; González-Vera, 2012; Nhu Ngoc Van and Morris, 2013; Figure 9B).

Fluorescent biosensors have been widely used by cell biologists to study the spatio-temporal dynamics and activities of enzymes in living cells and in real-time in physiological and pathological contexts, providing information which could not be obtained through traditional biochemical approaches. But these tools have also been largely implemented to biomedical applications, to highlight alterations in pathological disorders, and in drug discovery programmes to screen for, validate and characterize the efficacy of newly identified candidate drugs (Morris, 2012; Prével et al., 2014).

Likewise, CNTs constitute attractive biosensing devices for biomarker detection and imaging. SWNTs are indeed characterized by inherent photoluminescence between 650 and 1400 nm, which allows for deep penetration and imaging in biological tissues and organs with near- and infrared light (Smith et al., 2009). Unfunctionalized SWNTs possess low fluorescence stability, intensity, and biocompatibility. In contrast, surface functionalization, environmental changes, or interactions with target biomolecules affect SWNT fluorescence emission signals significantly (wavelength and intensity) (Chen et al., 1998; Moore et al., 2003), thereby making them well suited for fluorescence-based sensing applications (Boghossian et al., 2011; Iverson et al., 2013). Moreover, although SWNTs respond to changes in local dielectric function, they remain stable to photobleaching, therefore offering attractive opportunities for biomedical imaging applications (Barone et al., 2005).

For instance, SWNTs have been coupled to beta-D-glucose (Barone et al., 2005). SWNT/luciferase conjugates have been implemented for NIR detection of ATP in living cells (Kim et al., 2010). A chaperone sensor for nitrosoaromatics was engineered thanks to peptides immobilized on the CNT surface and further applied to image changes in peptide conformation associated with recognition of the molecular target through NIR photoluminesence (Heller et al., 2011; Figure 9C). Stabilization of SWNTs with genetically engineered M13 phage has been used for *in vivo* fluorescence imaging in deep tissues following intravenous injection (Yi et al., 2012). Likewise, non-covalent assembly of SWNTs with dye-labeled oligonucleotide yielded an optical biosensor of single-stranded DNA (ssDNA), fluorescence of which is quenched until the ssDNA target binds and releases the labeled oligonucleotide from the SWNTs (Yang et al., 2008; Figure 9D). Heteropolymers of SWNTs coated with a corona phase designed to recognize different metabolites (riboflavin, L-thyroxine and oestradiol) were used for NIR imaging of these compounds in space and in time in murine macrophages (Zhang et al., 2013). More recently, a label-free sensor of the troponin T was fabricated by immobilizing onto chitosan-wrapped nanotubes and used to detect this cardiac biomarker of acute myocardial infarction by NIR fluorescence (Zhang et al., 2014a).

#### Optical CNT biosensors for cancer detection

SWNTs-Indocyanine Green (ICG) conjugated with cyclic Arg-Gly-Asp (RGD) peptides to target alpha(v)beta(3) integrins were shown to serve as sensitive photoacoustic contrast agents following intravenous administration in to tumor-bearing mice, achieving subnanomolar sensitivity and 300 times higher photoacoustic contrast in living tissues than previously reported SWNTs (Zerda et al., 2010; Figure 9E).

SWNTs have been used to incorporate a fluorescent cyclin A binding motif derived from p21WAF1. A simple, selective and ultrasensitive fluorescence assay was developed for detection of cyclin A, a cell cycle regulator which is overexpressed in certain human cancers. The fluorescence of cyclin A binding peptide was quenched by energy transfer and electron-transfer processes but displayed fluorescence enhancement upon recognition and binding of cyclin A. This strategy demonstrated a nanomolar limit of detection of cyclin A and was thus proposed as a prognostic indicator of early stage cancer (Wang et al., 2010).

## Concluding remarks — challenges and outlook

With the growing demands of our society, in particular healthcare issues associated with aging of the population, next generation medical diagnostics require implementation of rapid, sensitive and cost-effective alternatives to the more traditional immunological assays currently used. Similarly, the detection of pathogens and toxic compounds in our environment constitute major threats and sensitive detection procedures are required to address concerns for our agriculture and global security.

An evergrowing number of biosensing devices and strategies have been developed since the Clark oxygen electrode in the 1950s. However, nanomaterials have clearly provided new and attractive platforms to engineer biosensors with enhanced sensitivity and performance. In particular, the structural, electronic, and optical properties combined by CNTs offer a wealth of opportunities to develop new generation nano-tools for biosensing applications, especially for probing disease markers.

The electronic properties of CNTs are extremely well suited for electrochemical, amperometric, impedometric, or field-effect transduction of signals, but their particular optical properties and their ability to penetrate readily through biological membranes also make them suitable candidates for the development of photoacoustic imaging sensors.

However, when proposing a biosensor, it is always recommended to take into consideration that nanoscale materials, although small, are not insignificant to leaving bodies; they can be sensed as intruders and consequently attacked. Therefore, issues related to their length, diameter, lifetime, stability, durability, mechanical properties, body adjustment, and toxicity must be evaluated by a scientific and systematic method. Indeed, when used in their pristine state, directly after synthesis, CNTs contain impurities and can have harmful effects. Nonetheless, when purified and surface-functionalized, their toxicity is drastically decreased and they represent optimal platforms for all kinds of applications. Besides, it was shown that continuous CNT fibers open a safe way to avoid the potential risk of CNT leaching, especially when used in implantable electrodes for *in vivo* testing (Zhu et al., 2010).

The high specific area of CNTs is a major advantage for biosensing applications, allowing for conjugation or complexation of an important number of receptor moieties, thereby contributing to increased recognition of targets or analytes, and consequently lowering the detection sensitivity threshold. CNTs have comparable dimensions to redox proteins and can be used as effective electrical wiring/connectors with redox enzymes. However, better control of the chemical and physical properties of the CNT biosensors is still needed. For example, the separation process for different types of CNTs, the miniaturization of the sensors and the *in vivo* stability need to be addressed to meet feature requirements. Besides, the nanowire morphology of CNTs enables the approach to the active centers of redox enzyme leading to fast and efficient electron transfers. In addition, cost-effective, large scale fabrication of CNT nanoelectrode arrays is an attractive option to produce greater currents than single nanoelectrodes, thereby circumventing requirement for expensive electronic devices, whilst improving the signal to noise ratio (Li et al., 2003). Hence, CNTs can overcome most disadvantages of a conventional electrochemical biosensor (including poor sensitivity and stability, low reproducibility, and large response times for electron transfer reactions) owing to their ability to undergo fast electron transfer and the resistance of CNT-modified electrodes to surface fouling, yielding ultrasensitive electrochemical sensors.

Importantly, the multifunctionalization of CNTs allows the design of biosensors for multiplexed detection of several biomarkers simultaneously. Although this constitutes a challenging objective, surface treatments, coating, and selective incorporation of chemical groups enable the functionalization of multiple biosensing moieties. Moreover, since functionalization has been shown to reduce the toxicity of CNTs, making them more biocompatible, and thereby addressing one of the major issues of these otherwise potent nanomaterials for cellular and *in vivo* applications, it clearly kills two birds with one stone.

CNTs therefore constitute versatile and multifunctional nanostructures which combine the potential to serve for diagnostic and therapeutic applications. Whilst they may serve as biosensors of cancer biomarkers with demonstrated characteristics of high sensitivity, reliability, and inexpensive microfabrication for cost effectiveness, they can also be loaded with anticancer drugs and used as therapeutic platforms in oncology. They may also be used for photothermic ablation of tumors and for photoacoustic molecular imaging of cancer cells. So, could CNTs be the Prince Charming the biosensor field was expecting?

### Conflict of interest statement

The authors declare that the research was conducted in the absence of any commercial or financial relationships that could be construed as a potential conflict of interest.

## Abbreviations

AFM: atomic force microscopy
AFP: autofluorescent Protein
BPE: bipolar electrode
CNT: carbon nanotube
DWNT: double-walled carbon nanotube
FET: field-effect transistors
FRET: fluorescence resonance energy transfer
GFP: green fluorescent protein
GOx: glucose oxidase
HER2: human epidermal growth factor receptor 2
MRI: magnetic resonance imaging
MWNT: multi-walled carbon nanotube
NIR: Near Infra-Red
OPN: osteopontin
PAABD: phosphor amino acid binding domain
PEI: polyethylenimine
PSA: prostate specific antigen
RBM: Radial Breathing Mode
ROS: reactive oxygen species
SSA: specific surface area
SWNT: single-walled carbon nanotube
TEM: transmission electron microscopy
VOC: volatile organic compounds.
